# Using Technology-Supported Transfer of Care Systems: Informing Good Practice Recommendations

**DOI:** 10.3390/pharmacy9010036

**Published:** 2021-02-11

**Authors:** Robert James, Efi Mantzourani, Cheryl Way, Alistair Gray, Melissa Burnley, Karen Hodson

**Affiliations:** 1 Cardiff School of Pharmacy & Pharmaceutical Sciences, King Edward VII Avenue, Cardiff CF10 3NB, UK; JamesR40@cardiff.ac.uk (R.J.); MantzouraniE1@cardiff.ac.uk (E.M.); 2 NHS Wales Informatics Service, 21 Cowbridge Road East, Cardiff CF11 9AD, UK; Cheryl.Way@wales.nhs.uk; 3 East Lancashire Hospitals NHS Trust, Royal Blackburn Teaching Hospital, Haslingden Road, Blackburn BB2 3HH, UK; alistair.gray@elht.nhs.uk; 4 Community Pharmacy West Yorkshire, Brooklands Court, Tunstall Road, Leeds LS11 5HL, UK; melissa@cpwy.org

**Keywords:** transfer of care, hospital discharge, community pharmacy, medicines optimisation, medicines adherence, healthcare technology

## Abstract

The Discharge Medicines Review (DMR) referral system, Refer-to-Pharmacy (RTP), PharmOutcomes and Help for Harry are UK transfer of care systems that aim to reduce the risks associated with hospital discharge. These systems use technology to facilitate the transmission of discharge information to community pharmacy, allowing community pharmacists to provide an adherence-support service. Despite the evidence that these systems benefit patient safety, there is a paucity of literature on their use. This study aimed to describe, compare and contrast these systems to highlight areas that could inform good practice recommendations. A rapid literature review was completed, and from the twenty-six sources of literature that were synthesised, three themes were identified for further exploration in semi-structured interviews with key informants: implementation, system attributes and stakeholder engagement. The key informants were purposively sampled for their role in the development and/or strategic implementation of each transfer of care system (*n* = 4). Audio recordings were transcribed ad verbatim and analysed both deductively and inductively. One interview was undertaken for each of the DMR, RTP and PharmOutcomes systems. Although all systems shared the same aim, differences were identified such as automated feedback for referrals, marketing strategies and practitioner accountability. Good practice recommendations suggested in this study could be applied to the future development of such systems.

## 1. Introduction

Patient transfer from hospital to the community is a process that is associated with many risks including medication errors, care discontinuity and subsequent hospital readmission [1]. Reducing the risk of medication-related harm during transfer of care, including hospital discharge, is considered an international priority as highlighted by the Medication Without Harm report from the World Health Organisation [2]. In the United Kingdom (UK), improving the transfer of information at hospital discharge has been prioritised by the King’s Fund, an independent organisation that aims to improve health and social care in England [3]. There have been numerous attempts to reduce the risks associated with transfer of care, typically focusing on specific aspects of the discharge process including electronic transmission of discharge information from hospital to community, post-discharge medicines reconciliation, and post-discharge support [4]. These attempts have been combined to different extents, creating more co-ordinated approaches called transfer of care systems. For many of these systems, community pharmacists are the healthcare professionals that provide post-discharge support [5]. This professional group can effectively identify and rectify post-discharge medication-related issues which could have resulted in patient harm, as evidenced by a recent systematic review [5]. International examples of these systems include the hospital-initiated Home Medication Review in Australia and the IBOM-1 protocol in the Netherlands, both involving post-discharge medicines adherence-support provided by community pharmacists [6,7]. Some of these systems use technology to facilitate the transmission of discharge information from hospital to community pharmacy.

In the UK, the National Health Service (NHS) is the umbrella term for the four health systems of the UK: England, Scotland, Wales and Northern Ireland. It is moving towards more technology-based solutions to improve patient care and to free healthcare staff capacity, as described by NHS England’s long-term plan and an independent review of technology utilisation in the NHS, the Topol Review [8,9]. Transfer of care systems are no exception and have been developed in the UK using technology. 

Since health is one of the devolved powers across these four devolved authorities of the UK [10], they are each responsible for their own health policies, including the digital strategy for health. As a result, there are multiple technology-supported transfer of care systems currently available in the UK, depending on locality. For example, in certain parts of England, PharmOutcomes, or Refer to Pharmacy or Help for Harry have been utilised, whereas in Wales, a national Discharge Medicines Review (DMR) system has been developed [11,12,13,14]. Each of these systems use technology to provide community pharmacists access to discharge information for their patients, allowing them to provide post-discharge medicines support to patients via a range of a commissioned services, which differ between countries. The evidence base supporting the use of some of these systems and the services they underpin is growing, with proven patient safety benefits, including identifying medication discrepancies and an association with a reduction in the risk of hospital readmission [13,15,16,17]. Despite the benefits of these systems, there is a paucity of published literature on their strategic implementation, processes and use.

With the increased emphasis on delivering healthcare embracing digital technologies, outlined in the Topol review in 2019 [9], combined with the renewed priority that the World Health Organisation placed on medication safety across transitions of care [18], it is imperative to construct the evidence base to support the development of transfer of care systems, both internationally and in the UK. 

In this study, we aimed to describe, compare and contrast the current UK technology-supported transfer of care systems to inform good practice recommendations.

## 2. Materials and Methods 

To achieve the study aims, a multi-method approach was used. This encompassed two methods which supplemented each-other. This is distinct to mixed methods research which uses both qualitative and quantitative methods, aiming to integrate the results to meet the research aims [19,20]. 

A rapid literature review, inclusive of grey literature, was performed to map the generic process of each system and to gather information about their provision and how they were strategically implemented. The literature identified through this approach was synthesised but lacked information regarding elements of the development and the strategic approach to implementation of the systems. To further explore the gaps identified from the literature, semi-structured key informant interviews were used [21,22]. 

### 2.1. Method 1: Rapid Literature Review

As there is a paucity of published literature on technology-supported transfer of care systems, the rapid literature review was developed to ensure all key literature was captured. Two stages were used to index published literature, but also to include a more specific search for grey literature. The first stage was a structured literature search in databases that index journals related to healthcare research. The information gathered from this approach was then supplemented with a targeted literature search in grey literature sources such as YouTube and National Institute for Health and Care Excellence (NICE), using more specific search terms.

#### 2.1.1. Search Strategy and Resources

The search strategy was developed iteratively to index all resources relevant to UK technology-supported transfer of care systems. The authors were aware that there were no well-established electronic transfer of care systems developed before the DMR service in 2011, and therefore, the search was restricted to the last ten years to ensure the development of any active systems were included. The exclusion criteria were articles with no relevance to UK transfer of care systems. The search strategy for both stages of the rapid literature review is described in Table 1. 

#### 2.1.2. Quality Assessment

No formal tool was used to assess the quality of indexed articles before inclusion, as the aim was to describe, compare and contrast the different systems. 

#### 2.1.3. Synthesis of Literature

The final literature identified from both stages of the rapid literature search was combined and then synthesised to describe the generic process for each technology-supported transfer of care system and to compare them. To allow the timely synthesis of literature, one author performed the synthesis (RJ) and discussed frequently with another two authors (KH and EM), a method supported by the literature [23]. Once the literature had been synthesised and the systems were compared, a number of areas were identified where not enough information was retrieved to allow an in-depth comparison of the systems. It was therefore decided to supplement the literature review with key stakeholder interviews. 

### 2.2. Method 2: Key Stakeholder Interviews (29th May to 8th July 2019)

The semi-structured key informant interviews were performed either face-to-face or by telephone. A generic qualitative approach was used to supplement the information identified from the literature review [21,22]. The interview schedule (Appendix A) sought clarity around the description of each system, and explored concepts around the attributes of each system, their referral processes and the approach to their strategic implementation. To improve transparency of reporting, the Standards for Reporting Qualitative Research has been completed and attached as Appendix B [24].

#### 2.2.1. Study Approvals

Ethical approval was granted by the Cardiff School of Pharmacy & Pharmaceutical Sciences Ethics Committee on 8 March 2019 (1819-11). As some participants were NHS employees, the study was registered with their employing healthcare authority where required.

#### 2.2.2. Participants and Recruitment

The population were individuals who were involved with the development and/or strategic implementation of a UK technology-supported transfer of care system. The sampling frame was identified from the literature, and key informants were purposively sampled for each of the four systems [11]. 

One participant for each system was considered appropriate as the aim was not to reach saturation, but to further describe aspects of each system.

All four participants were invited to participate by email with an attached participant information leaflet and consent form. A reminder email was sent to non-responders after two weeks.

#### 2.2.3. Data Collection and Analysis

Interviews were audio-recorded with consent, transcribed ad verbatim using Microsoft Word and identifying information was removed to ensure confidentiality [25]. A mixed thematic analysis was performed using NVivo (version 11) [25]. Firstly, the data were deductively analysed, searching for concepts identified from the literature. The data were then analysed inductively, as described by Braun and Clarke [26], to construct themes from any other information that was not captured in the deductive analysis. RJ and KH independently coded the data and a meeting was held to confirm that there was inter-coder reliability [21].

#### 2.2.4. Reflexivity

RJ is a practising community pharmacist in Wales with experience using the DMR referral system. This could introduce bias as he had preconceived ideas of barriers and facilitators to engaging with the DMR referral system. To mitigate the effects of potential bias, a reflective diary was kept through the process of collecting data, transcribing and data analysis. This allowed reflection on the potential effect of bias at every stage of the research process, allowing interpretation to be data-driven.

## 3. Results

### 3.1. Method 1 Results: Rapid Literature Review

The rapid literature review was performed between 8 October and 25 November 2018. 

**Figure 1:**
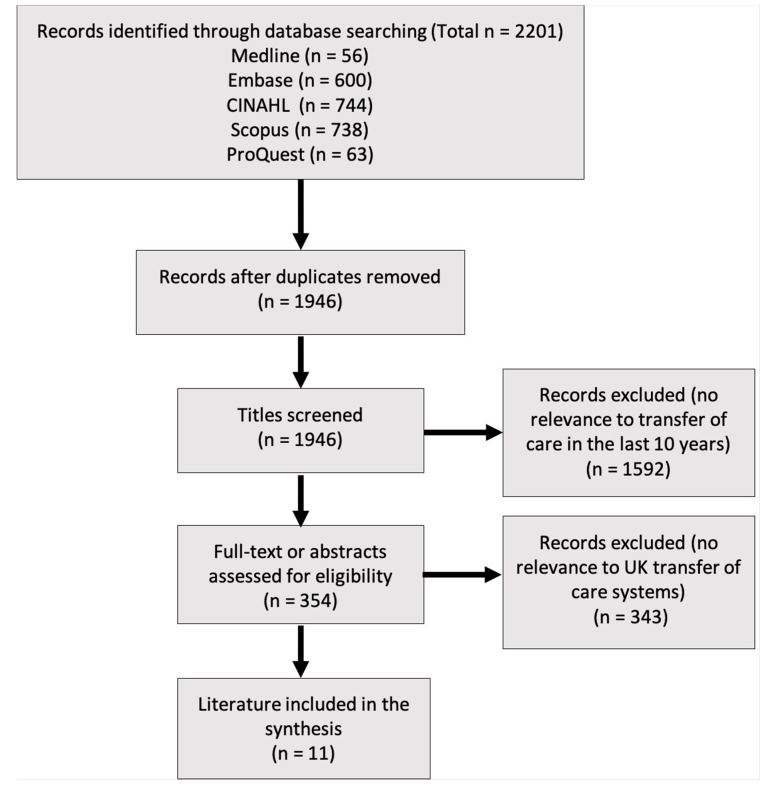
PRISMA flow diagram describing the refinement of the structured literature search.

The structured literature search identified eleven relevant results for inclusion. The number of hits and refinement of the literature is described in a PRISMA flow diagram in Figure 1. No additional literature was discovered through reference screening. 

Fourteen additional sources were identified from the targeted literature search. One further source was identified through reference screening of the targeted literature search results. A summary of the literature can be found in Supplementary Materials Table S1. The twenty-six sources of literature that were identified from this review were synthesised to first describe the generic processes for each system and to then identify areas that contrast. Three broad concepts were identified from the literature in which the systems contrast: implementation, stakeholder engagement and system attributes.

#### 3.1.1. Transfer of Care System Processes

The DMR referral system, Refer-to-Pharmacy and PharmOutcomes used IT platforms to facilitate community pharmacist access to a patient’s discharge information, whereas Help for Harry used fax transmission. This information was transferred to support the community pharmacist in providing post-discharge support. For the DMR referral system and Help for Harry, this information was specifically to support the completion of an adherence-support service. Refer-To-Pharmacy and PharmOutcomes referrals could be for adherence-support services or could be for information only, for patients who have their medicines dispensed into compliance aids, for care home residents and for the provision of other post-discharge support services, such as smoking cessation [27,28]. 

**Figure 2:**
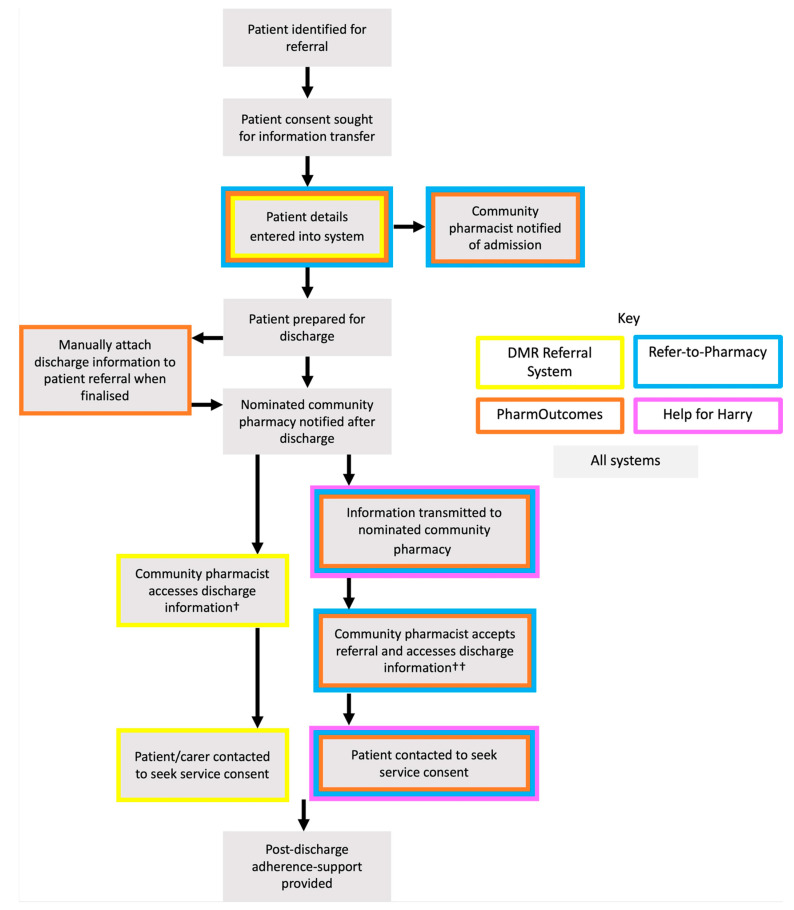
Process map for UK technology-supported transfer of care systems. Footnotes denoted by † are used to further describe selected aspects of the figure. † Discharge information is stored within the Welsh Care Records Service and accessed via the all-Wales shared community pharmacy IT platform, Choose Pharmacy. Only the nominated pharmacy is notified of discharge, but discharge information can be accessed by any pharmacist with patient consent. †† Information available only to the nominated pharmacy.

Synthesis of the literature was used to describe the transfer of care system processes as shown in Figure 2 [11,28,29,30].

#### 3.1.2. Identifying Differences

Some features of the transfer of care systems that contrast are described in Table 2, whilst areas requiring more detailed explanation are described below.

##### Notifications

Each system notifies the community pharmacy about the patient’s hospital status. Refer-to-Pharmacy and PharmOutcomes send a notification when a patient is referred to inform the pharmacy that their patient is in hospital, and at discharge to inform them that the patient is going home [32,36]. The DMR referral system and Help for Harry only notify the community pharmacy of the patient’s discharge.

Help for Harry sends notifications by fax where all the other systems send anonymised notifications electronically [11,30]. These anonymised notifications contain no patient details but inform the community pharmacist that they have discharge information ready to access. DMR referral notifications can be sent through the NHS email network or via the all-Wales shared community pharmacy IT platform, Choose Pharmacy. Refer-to-Pharmacy and PharmOutcomes notifications can also be sent through the NHS email network, and also to personal email addresses [27,30,37]. A USB device called a Pharmalarm can be purchased for use with PharmOutcomes, which flashes on receipt of notifications [32].

##### IT Interoperability

The extent to which the transfer of care systems were integrated with existing community and hospital IT systems varied. Interoperability with either of these IT systems would mean that practitioners did not have to manually transcribe discharge information from their IT system into the transfer of care system, or vice-versa. The DMR referral system and Refer-to-Pharmacy are fully interoperable with hospital IT systems, allowing seamless referrals from the hospital without requiring additional data entry [28,30]. The majority of hospital IT systems do not integrate with PharmOutcomes, requiring the patient information to be manually transcribed into the system. Some CCGs have negotiated individually with PharmOutcomes such that it is interoperable with their systems, for an additional cost [29,32]. Refer-to-Pharmacy and PharmOutcomes referrals are not interoperable with community pharmacy IT systems. In practical terms, this means that once a community pharmacy has received a referral, they will have to enter the patient details separately into their relevant systems for providing advanced pharmacy services, such as the NMS and dMUR [27,37]. In contrast, the DMR referral system is fully interoperable with Choose Pharmacy. All the fields for completing a DMR will be auto-filled, requiring no manual transcription [30]. Help for Harry uses fax transmission; therefore, it is not interoperable with either hospital or community pharmacy IT systems [11].

##### Management of Patient Consent

Consent for all transfer of care systems is required in two stages. The first stage of consent is for making the discharge information available for community pharmacists and then consent is required for the provision of the follow-up service [17,30]. Consent for information transfer is sought verbally by hospital pharmacists or pharmacy technicians. For PharmOutcomes and Refer-to-Pharmacy this will be sought on admission, allowing both admission and discharge notifications to be generated [28,38]. However, consent can be sought at any time before discharge and a discharge notification will still be generated. Since the DMR referral system and Help for Harry do not generate admission notifications, consent only needs to be taken before the point of discharge [11,17].

Refer-to-Pharmacy and the DMR referral system allow transfer of information for patients who cannot consent when the referring professional believes it is in their best interests to do so. This is acknowledged by a flag in the systems [30,36].

Refer-to-Pharmacy has an inbuilt video to support the consent process. This video describes the process and the benefits of a referral to help inform patients of the availability of post-discharge support [28]. 

##### Patient Referral Eligibility and Screening

For patients to be referred from one of these transfer of care systems, they must meet the patient inclusion criteria for the relevant post-discharge support service in their respective locality. Each of these services has a distinct service delivery model, as described in Table 3 [11,17,39,40]. 

When identifying a patient for post-discharge medicine support through Refer-to-Pharmacy or the DMR referral system, the referring practitioner is required to select the patient’s eligibility criteria from a drop-down menu [41]. The system will not allow a patient to be referred unless one of the criteria is met. PharmOutcomes does not require a practitioner to specify why patients are eligible, even though the criteria for referral are shown on the system’s screen [29]. 

In addition to selecting eligibility criteria, Refer-to-Pharmacy and PharmOutcomes require the practitioners to stipulate a reason for the referral, for example if they believe the patient would benefit from a discharge MUR (dMUR), NMS or post-discharge medicines counselling [28,29]. As the DMR referral system is only used in Wales and has been developed specifically to refer patients to the DMR service, there is no additional requirement for a referral reason to be stated.

##### System Enabled Dissemination of Service Outcomes 

Refer-to-Pharmacy sends an automated email to the referring practitioner once a referral has been actioned. This email contains information about the outcomes of the service, including what post-discharge service was provided and whether the referral prevented a discrepancy, and saved time or money. PharmOutcomes provides the referring practitioner access to data regarding whether the referral was accepted and if so, which post-discharge service was completed [27,38]. Uniquely, PharmOutcomes will also send a copy of this report to the patient’s GP surgery if they have an email address registered on the system [29]. The DMR referral system and Help for Harry provide no routine feedback to the referring practitioner.

### 3.2. Method 2 Results: Key Informant Interviews

Three of the four key informants were interviewed between 29 May and 8 July 2019. The informant for Help for Harry was unable to participate due to a change in their job role. No further key informants could be identified due to a lack of published information about the system. Two interviews were completed by phone and one was completed face to face. These interviews lasted between 45 and 75 min.

Three broad concepts, identified from the literature, were deductively explored in the data: implementation, stakeholder engagement and system attributes. When the data were analysed inductively, no further superordinate themes were identified, but sub-themes were identified which assisted the organisation and analysis of the data. Through this section, system similarities and differences identified through the interviews are described.

#### 3.2.1. Theme 1. Implementation

The key informants identified ways in which the implementation of the systems contrasted. These areas include community pharmacist engagement, marketing strategies, collaboration, dedicated staff and piloting. 

##### Community Pharmacist Engagement

The key informants for two systems discussed how they purposively engaged with community pharmacists during system implementation, ensuring that they were aware of the system, knew how to action referrals and were able to receive them.

“There were some people who, again human factors, took a few phone calls to say “please fill the form in”, “oh yeah we’ll do it now we’ll do it now” and of course they didn’t so we had to phone them back so again this took a few months sort of to actually get around to filling out the form to receive the referrals”.

(P2)

##### Marketing Strategies

For the implementation of one system, a pre-determined marketing strategy was used, including regular newsletters to interested parties, speaking on speaker circuits and creating instructional videos on platforms such as YouTube.

“I wanted to create some sort of marketing strategy, so I got onto speaker’s circuit at various conferences, started sending out a newsletter to interested parties to keep them informed of developments and that helped sort of create an awareness of what we were actually doing”.

(P2)

##### Collaboration

All key informants discussed the importance of collaborating with local professional organisations that support the provision of community pharmacy services, such as Community Pharmacy Wales and Local Pharmaceutical Councils (LPCs). These organisations helped engage with stakeholders through implementation.

“So then as an LPC, we supported all the contractors, so we spent time showing them how to use [transfer of care system], how to set up accounts, how to use the system so by pulling down a report we can see who’s not regularly engaging and spending time with them making sure they do engage”.

(P3)

##### Dedicated Staff

Key informants for two systems discussed the importance of employing staff specifically to implement the system. These staff typically organised marketing, developed materials to support implementation and engaged with stakeholders.

“Yeah yeah well that [funding] paid for me which really helped sort the meetings at the hospital, ring the pharmacies, develop guides, develop the [transfer of care system] cos all that takes time really and so we have engagement events so which is a lot of work we need to get the service up and running”.

(P3)

##### Piloting

Each system was piloted differently in their locality. One system was piloted with patients who have their medication dispensed into compliance aids because the transmission of discharge information for this population was already common by fax. Another system was piloted geographically, initially implemented in approximately forty-two pharmacies and then slowly implemented across the rest of the locality. The third system didn’t undertake a pilot, implementing the system to the entire locality overnight instead.

“No, no, no [we didn’t pilot], it was rolled out across our trust’s footprint in a big bang way”.

(P2)

#### 3.2.2. Theme 2. System Attributes

The key informants highlighted differences between the attributes of each system, including IT interoperability, referral prompts and referrals to alternative practitioners.

##### IT interoperability

IT interoperability was discussed by all of the participants. IT interoperability, between the transfer of care system and both hospital and community IT systems, was considered a facilitator to system engagement.

“It [the transfer of care system] populates the form for you, that saves them [community pharmacists] time as well so all they’ve got to go is “is it the same?”, if it’s different, what’s different and tick boxes, so we’ve tried to make it as easy as possible for them. So, I think it’s probably removed a number of the barriers”.

(P1)

One participant described how the lack of interoperability and integration between their system and the hospital IT system was a significant barrier for system engagement, as staff would have to log into the separate application and manually transcribe all the information.

“It’s still an extra step for them [referring practitioners] I think, it’d be better if it was integrated into the hospital IT system somehow cos we use a web-based platform and although it’s a quicker system than using a fax for the trust except for the departments in the hospital it’s still a lot of logging in and that’s what they said they’d use it if it was integrated”.

(P3)

##### Referral Prompts

One participant discussed how their system had inbuilt prompts to encourage the staff to refer patients for post-discharge support when appropriate. This prompt appears on the computer screen when a staff member records a patient’s drug history. This was perceived as being beneficial for system engagement.

“It [transfer of care system] prompts to make a referral if they’re a blister pack patient or a care home resident so we’re pretty good at making those referrals”.

(P2)

##### Referrals to Alternative Practitioners

Two participants discussed how their systems have the functionality of referring patients to different healthcare practitioners. Examples of these include local anticoagulation clinics, mental health services and domiciliary support teams.

“We also send referrals to what we called a medicines support team so a domiciliary pharmacy support service for people from [CCG name] so that’s to arrange home visits for people who are housebound or can’t easily access community pharmacy services and they can visit them in their home”.

(P2)

#### 3.2.3. Theme 3. Stakeholder Engagement

The key informants discussed methods by which stakeholder engagement with the system is encouraged, including accountability for referrals, routine feedback to hospitals and staff training tools.

##### Accountability for Referrals

All of the key informants considered that keeping staff accountable for referrals was a facilitator for system engagement. Staff using two systems kept community pharmacists accountable for actioning referrals by creating weekly reports for referrals which had not been actioned, following up those pharmacies by phone to identify any issues.

“I’ve seen some of the other platforms go live and they’ve had no support for community pharmacists and if you’ve got no-one pulling down a report to see which pharmacies are doing it, it just gets forgotten about, the pharmacists don’t know how to use the system and then it just falls, falls apart”.

(P3)

Staff using one system kept the hospital pharmacy staff accountable for referring patients. They achieved this by giving feedback to pharmacy teams when eligible patients had been discharged without a referral. 

##### Feedback of Service Outcomes to Hospitals

The participants discussed the varying levels of feedback that their system provides to referring practitioners. One system provides automated and anonymised feedback to the referring practitioner by email, including information on the outcomes of the referral. This information includes what post-discharge support was provided to the patient, whether medication discrepancies were prevented and whether the referral saved money or time. This was considered a facilitator for system engagement as the practitioner would see the benefit and outcome of their referral.

One participant discussed how their system did not have automated feedback to the hospital, but the LPC held regular meetings with the hospital pharmacy department to share information such as the number of referrals made and their outcomes.

“We have regular meetings with the hospital as well so they can see what the pharmacy is doing, y’know it’s not just going into the ether like a fax was, they can see all the feedback and they’re loving seeing all the data that pharmacy’s doing and they’re like “let’s keep going cos, let’s send more referrals, who else could we refer because pharmacy is really engaging with this so”. So they’re really enjoying it as well, they’re really happy”.

(P3)

The third system had not yet integrated routine feedback for referring practitioners, but the participant considered that it would be a facilitator for system engagement and suggested that it was a planned improvement for the near future. 

##### Staff Training Tools

The key informant for one system described that they have developed a training quiz to help improve engagement with referrals by increasing staff knowledge.

“So, I thought I’ll do a [transfer of care system] quiz and that’s been used by the staff to sort of get them into understanding why someone is eligible for referral”.

(P2)

These quizzes are developed from cases where eligible patients were not referred, giving targeted training to improve knowledge of which patients are eligible for referrals. 

## 4. Discussion

This study used a multi-method approach to describe, compare and contrast UK technology-supported transfer of care systems. Concepts around development and/or strategic implementation were explored, and areas were highlighted that could form the evidence base in the development, implementation or improvement of similar systems.

One difference highlighted by the study arose from the criteria that the different systems use to screen the eligibility of patients to receive post-discharge support services. A system should ideally facilitate support to those patients most at risk of post-discharge medicines mismanagement, increasing the equity of access to healthcare. However, Table 3 illustrates that at present some of the systems cannot refer patients to services if they are housebound and cannot provide certain services direct to carers, who may be the person managing the patient’s medicines. It is suggested that more flexible criteria be allowed, to ensure that those patients most at risk of medication harm where there is a transition of care can be assured of post-discharge medication support [42,43,44,45]. 

Since the completion of this study, post-discharge support services in England have changed. As of April 2021, the MUR service is being decommissioned in England [35], and a new Discharge Medicines Service was commissioned in July 2020 [17]. No new IT system has been developed for this service and it is suggested that referral to community pharmacies be done via any secure electronic platform, such as PharmOutcomes, Refer to Pharmacy or secure NHS email [46]. The eligibility criteria for referral for this new service can be defined by NHS trusts, but the published toolkit supporting its implementation described a wide range of broad potential criteria, allowing increased flexibility [47].

Another important aspect arising from the results is in relation to the interoperability of the transfer of care systems with wider healthcare IT, a key enabler for the more widespread adoption of IT. This is one of the priorities for NHS England, as outlined in the NHS five-year plan; to increase the level of IT utilisation for improvements in care continuity, including patient discharges from hospital [48]. The Topol review also independently determined that there is a need to increase IT provision and integration in the NHS [9]. Although there is limited research surrounding the benefits of IT interoperability, systems that are less interoperable will have an inherently higher risk of transcription errors and will be more time-consuming, and therefore, are likely to disrupt the workflow of service users. This challenge was described by participants in the current study, where having to use a system without extensive IT interoperability was considered as “an extra step” when referring patients for post-discharge support. The non-adoption, abandonment, scale-up, spread, sustainability (NASSS) framework asserts that if the technology significantly disrupts workflow, it is less likely to be adopted [49]; therefore, it is crucial that transfer of care systems should be interoperable with both hospital and community IT systems.

All systems included in this study had a two-stage process for obtaining patient consent, in line with clinical governance. However, patient consent is often considered a barrier to primary care services and has specifically been identified as a barrier to the DMR service through previous studies, including interviews with community pharmacists [17,50]. A system feature described by one of the participants was the integration of videos to support the consent process. Since the evidence for the use of multi-media consent aides in healthcare is growing, it would be prudent to adopt video consent aides to assist practitioners in gaining patient consent [51,52,53].

A key difference between the systems was found to be related to mechanisms for notifying community pharmacists that one of their patients had been discharged from hospital, and hence is in need of post-discharge support. It has been reported in the literature that a lack of awareness for their patients’ discharge is a key barrier for community pharmacists providing post-discharge support to their patients [17], increasing patient risk by potentially supplying patients with outdated prescriptions and also increasing potential medicines waste. Notification systems can be a solution to this problem, with alerts that should be visible and easily accessible to facilitate community pharmacist awareness of a patient’s hospital status. Different notification methods can help improve visibility, as identified in this study, such as the Pharmalarm, and the allowing of email notifications to be sent to personal NHS email addresses as well as the pharmacy’s work email address. The use of only a personal email address for these notifications could prove troublesome, as other pharmacists working in the same premises would not have access. 

At the time of data collection, only one of the systems studied enabled the automatic dissemination of the outcomes of any post-discharge support interaction between the patient and their community pharmacist back to the hospital practitioner who initiated the referral. Previous studies with hospital pharmacists who use two UK technology-supported transfer of care systems reported that such lack of any further feedback feels like referring patients into a “*black hole*” [17,18]. Normalisation Process Theory describes how the implementation and embedding of an innovation is more likely when stakeholders are able to reflect on its effectiveness [54]. The routine automated feedback described in the current study would help fulfil this criterion, allowing practitioners to see that their referral was actioned, and the outcome of the post-discharge service. More systems are increasing hospital staff access to post-discharge service outcomes: as of April 2020, the DMR referral system increased its functionality to automatically upload the outcomes of each DMR service to the shared clinical record in Wales, accessible by hospital pharmacy staff. During the current COVID-19 pandemic, this feedback is also accessible by GPs [55].

Aspects of the strategic implementation of the different technology-supported transfer of care systems, described in this study, are supported by a recent systematic review of the factors affecting the implementation of electronic interventions in healthcare [56], and can therefore be recommended as good practice. Examples include the use of dedicated staff or champions to support the implementation of technology, and suggest that implementation should be planned, ensuring that service users are adequately trained and stakeholders are informed of developments in a timely manner. The review also suggests that incremental piloting is beneficial for the implementation of technology, in contrast to the system that was implemented to the entire locality overnight [56].

### Limitations

Since the end of the data collection period for this study, further developments in transfer of care systems were introduced in the UK. Even though these developments did not form part of the results, they have been referenced and included in the discussion section. The use of grey literature provided extensive information for the description and comparison of the UK technology-supported transfer of care systems. Although these sources are not peer reviewed, the areas of good practice were supported by background literature before being used to draft recommendations. 

Despite the fact that the primary researcher is a practising community pharmacist in Wales, and this could potentially introduce bias in data collection and interpretation, care was taken to avoid this at every stage of the research process through the use of a reflective diary and different authors being involved in the quality assurance of data analysis. 

Despite the small sample size in the qualitative arm of this study, the authors do not believe that any bias was introduced. The purpose of the key informant interviews was to supplement the rapid literature review with the further description of elements of development or strategic implementation, rather than their perceptions of the systems, so one participant for each system was considered appropriate. This study focused on UK technology-supported transfer of care systems, and as such, the transferability of this study is limited to the UK. However, some of the good practice may be applicable to the development of international systems. 

## 5. Conclusions

This is the first study to describe, compare and contrast current UK technology-supported transfer of care systems. Based on the discussions outlined in this paper, the following timely recommendations are suggested for the development, adaptation and strategic implementation of technology-supported transfer of care systems:

1. Pre-plan implementation strategies with dedicated staff, focussing on stakeholder engagement;
2. Flexible notification systems should be developed to inform community pharmacists of patient admission and discharge, including email and USB device notifications;
3. Produce content such as videos to support patient consent for information transfer;
4. Develop methods to keep hospital and community practitioners accountable for referrals;
5. Develop interoperability with both hospital and community IT systems to make referrals seamless;
6. Ensure post-discharge adherence-support services have broad eligibility criteria.

The use of these technologies is likely to be adopted more widely internationally with the World Health Organisation’s focus on improving medication safety during transitions of care, and in the UK with the recent announcement in England of the Discharge Medicines Service [18,57]. Further work to explore stakeholder perceptions of these systems would provide more evidence of service users’ perspectives.

## Figures and Tables

**Table 1:** Search strategy for literature review to describe the implementation and processes of UK technology-supported transfer of care systems.

Eligibility Criteria	
Published between January 2009 and November 2018 Published in English Relates to UK technology-supported transfer of care systems	
**Structured Literature Search**	
Databases Searched	Search Terms
MEDLINE CINAHL EMBASE	“Patient transfer” OR “Care adj3 transfer” OR “Care adj3 transition” OR “hospital discharge” OR “information adj3 transfer” **AND** **“**Pharmac *” “community pharmacy” “community pharmacist”
SCOPUS PROQUEST	“Care w/3 transfer” OR “hospital discharge” OR “information transfer” OR AND “community pharm *” Including only literature indexed under ‘Nursing’, ‘Health professions’, ‘Medicine’, ‘Psychology’, ‘Social Sciences’, ‘Toxicology and Pharmacology’ and ‘Economics’ categories.
**Targeted Literature Search**	
Pharmaceutical Journal International Pharmaceutical Federation Clinical Pharmacist National Institute for Health and Care Excellence (NICE) Royal Pharmaceutical Society Pharmaceutical Service Negotiating Committee (PSNC) Community Pharmacy Wales East Lancashire NHS Trust YouTube	Hospital discharge Refer-to-Pharmacy PharmOutcomes Discharge Medicines Review DMR Help for Harry

**Table 2:** Key similarities and differences between the UK technology-supported transfer of care systems.

	DMR Referral System	Refer-to-Pharmacy	PharmOutcomes	Help for Harry
Location [11,17,31,32]	Wales (only available from hospital wards that use the all-Wales electronic discharge IT system)	East Lancashire and Blackburn with Darwen Clinical Commissioning Groups (CCG)	Devon, Hampshire and Isle of Wight, North of Tyne, Thames Valley, Buckinghamshire, Cornwall and Isles of Sicily CCGs	Derbyshire NHS Trust
System developers [11,30,31,33]	NHS Wales Informatics Service	Webstar-Health in conjunction with East Lancashire CCG	Pinnacle Health Partnership LLP	Derbyshire NHS Trust
Funding for IT infrastructure [11,17,33]	Funded centrally by the Welsh Government.	Funded between CCGs and NHS Trust.	Funded by CCG. Payment varies depending on how much functionality is needed.	No IT infrastructure costs. The fax machine is supplied by the individual hospital.
The extent of information transfer [28,30]	Discharge medication information only.	Clinical information and discharge medication information.	Discharge medication information only.	Discharge medication information only.
Adherence-support service [11,30,31]	DMR service †	Medicines Use Review (MUR) or New Medicines Service (NMS) ††	MUR or NMS	MUR or NMS
Post-discharge service payment to pharmacy contractor [17,34,35]	GBP 37 per DMR	GBP 20–28 per NMS GBP 28 per MUR	GBP 20–28 per NMS GBP 28 per MUR	GBP 20–28 per NMS GBP 28 per MUR

† The DMR service is a commissioned community pharmacy service in Wales which consists of medicines reconciliation (part one) and adherence-support (part two). †† MUR and NMS are community pharmacy services that are commissioned in England and that provide medicines adherence-support. Payment for the NMS service is graduated; contractors will be paid higher rates per service if they have provided more NMSs in that financial year relative to the maximum of four hundred.

**Table 3:** Service model delivery for post-discharge adherence-support services.

Patient Inclusion Criteria		
DMR	NMS	Discharge MUR
Medication change in hospital	New medication for type 2 diabetes, COPD, asthma, hypertension or anticoagulation	Taking two or more medications
More than four medications		Medication change in hospital
The patient has their medication dispensed into a compliance aid		
Professional judgement		
**Location of review**		
In the pharmacy Telephone consultation In the patient’s home (with commissioner’s permission)	In the pharmacy Telephone consultation	In the pharmacy Telephone consultation In the patient’s home (with commissioner’s permission)
**Who can receive the review?**		
Patient or carer	Patient only	Patient only

## Data Availability

The data presented in this study are not available on request from the corresponding author. This is due to the specific roles of key informants possibly being identified from the transcripts. Consent from the participants was obtained for the use of anonymised quotations from the interviews and not for access by a third party to the whole transcripts.

## Supplementary Materials

The following are available online at https://www.mdpi.com/2226-4787/9/1/36/s1, Table S1: Brief summary of literature.

## Appendix A

(1) Could you explain a bit about the history of DMR/Refer-To-Pharmacy/Pharmoutcomes/Help for Harry?
  - Why was it set up?
(2) Could you please take me through a step by step process of the service from identification of patients to post-discharge follow-up
(3) How is patient consent managed throughout the service?
  - Hospital consent (what is this for?)
  - Community consent (what is this for?)
(4) What data are routinely collected through each service?
  - Medication names
  - Number of discrepancies
  - Outcome of referral
  - Demographics
(5) How many pharmacies currently provide this service? (clarify if this is increasing)
  - How many did it start with, as a pilot?
  - Is this still increasing?
  - How have you managed to get pharmacies on board?
(6) Through research on DMRs, it was found that many hospital staff felt that they initiate the scheme but see no end-product. What feedback is routinely provided to hospital staff?
  - How is this recorded?
(7) How do community pharmacists receive notification that a patient has been discharged from hospital? (clarify whether personal/NHS email, etc.)
  - Has this changed since inception of the service?
  - Do you see any issues with these methods?
  - Any additional notifications provided? (admission)
(8) What do you consider are the barriers to the provision of RTF/DMR/PharmOutcomes?
  - Have these changed over time?
(9) What do you consider are the facilitators to the provision of RTP/DMR/Pharmoutcomes/Help for Harry?
  - Have these changed over time?
(10) What, if any, improvements or advances are planned in the foreseeable future for this service?
  - Are changes in services based on service evaluations?
  - What further service evaluations are planned and how do you hope these will implement further change?

Do you have any further comments or information you think would be useful?

[Prompt—summarise all key points to ensure accurate data collection].

Thank you again for your time.

## Appendix B

**Table A1:** Summary for Reporting Qualitative Research items and their corresponding lines within the text.

Summary for Reporting Qualitative Research Item	Corresponding Line(s)
Item 1. Title: Concise description of the nature and topic of the study. Identifying the study as qualitative or indicating the approach (e.g., ethnography, grounded theory) or data collection methods (e.g., interview, focus group) is recommended.	2–3
Item 2. Abstract: Summary of key elements of the study using the abstract format of the intended publication; typically includes background, purpose, methods, results, and conclusions.	13–28
Item 3. Problem Formulation: Description and significance of the problem/phenomenon studied; review of relevant theory and empirical work; problem statement.	32–73
Item 4. Purpose or research question: Purpose of the study and specific objectives or questions.	70–76
Item 5. Qualitative approach and research paradigm: Qualitative approach (e.g., ethnography, grounded theory, case study, phenomenology, narrative research) and guiding theory if appropriate; identifying the research paradigm (e.g., post-positivist, constructivist/interpretivist) is also recommended; rationale.	115–118
Item 6. Researcher characteristics and reflexivity: Researchers’ characteristics that may influence the research, including personal attributes, qualifications/experience, relationship with participants, assumptions, and/or presuppositions; potential or actual interaction between researchers’ characteristics and the research questions, approach, methods, results and/or transferability.	143–149
Item 7. Context: Setting/site and salient contextual factors; rationale.	126–134
Item 8. Sampling strategy: How and why research participants, documents, or events were selected; criteria for deciding when no further sampling was necessary (e.g., sampling saturation); rationale.	126–134
Item 9. Ethical issues pertaining to human subjects: Documentation of approval by an appropriate ethics review board and participant consent, or explanation for lack thereof; other confidentiality and data security issues.	122–125
Item 10. Data collection methods: Types of data collected; details of data collection procedures including (as appropriate) start and stop dates of data collection and analysis, iterative process, triangulation of sources/methods, and modification of procedures in response to evolving study findings; rationale.	135–142
Item 11. Data collection instruments and technologies: Description of instruments (e.g., interview guides, questionnaires) and devices (e.g., audio recorders) used for data collection; if/how the instrument(s) changed over the course of the study.	Appendix A 122–124
Item 12. Units of study: Number and relevant characteristics of participants, documents, or events included in the study; level of participation.	126–134
Item 13. Data processing: Methods for processing data prior to and during analysis, including transcription, data entry, data management and security, verification of data integrity, data coding and anonymization/de-identification of excerpts.	135–142
Item 14. Data analysis: Process by which inferences, themes, etc., were identified and developed, including the researchers involved in data analysis; usually references a specific paradigm or approach; rationale.	135–142
Item 15. Techniques to enhance trustworthiness: Techniques to enhance trustworthiness and credibility of data analysis, (e.g., member checking, triangulation, audit trail); rationale.	141–142
Item 16. Synthesis and interpretation: Main findings (e.g., interpretations, inferences, and themes); might include development of a theory or model, or integration with prior research or theory.	265–409
Item 17. Links to empirical data: Evidence (e.g., quotes, field notes, text excerpts, photographs) to substantiate analytic findings.	265–409
Item 18. Integration with prior work, implications, transferability, and contribution(s) to the field: Short summary of main findings, explanation of how findings and conclusions connect to, support, elaborate on, or challenge conclusions of earlier scholarship; discussion of scope of application/generalizability; identification of unique contribution(s) to scholarship in a discipline or field.	410–485
Item 19. Limitations: Trustworthiness and limitations of findings.	486–503
Item 20. Conflicts of interest: Potential sources of influence or perceived influence on study conduct and conclusions; how these were managed.	530
Item 21. Funding: Sources of funding and other support; role of funders in data collection, interpretation, and reporting.	527–528
