# Supplementary material for: Using Technology-Supported Transfer of Care Systems: Informing Good Practice Recommendations
Source: Pharmacy (Basel). 2021 Feb 11;9(1):36. doi: 10.3390/pharmacy9010036 (PMC8005999; doi:10.3390/pharmacy9010036)
Supplement: Supplementary file 1 [file pharmacy-09-00036-s001.pdf]

Table S1: Brief summary of literature gathered from the structured literature search

Author(s)	Publisher	Literature type	Year	Literature Title	Brief Summary
Mantzourani, E et al	International Journal of Pharmacy Practice	Abstract	2014	Information required by community pharmacists to complete a Discharge Medicine Review for patients when they are discharged from hospital	Background information about the DMR service. Describes community pharmacist perceptions of what information is required to perform a DMR, and how they would like to receive it.
Hodson, K et al	International Journal of Pharmacy Practice	Abstract	2014	Evaluation of the Discharge Medicines Review Service in Wales: Content analysis of Discharge Medicines Reviews	Background information about the DMR service. Describes the uptake of the DMR service and its impact on the health economy and patient safety.
Hodson, K et al	International Journal of Pharmacy Practice	Abstract	2014	Evaluation of the Discharge Medicines Review Service in Wales: community and hospital pharmacists' views	Background information about the DMR service. Explores community pharmacist and hospital pharmacist perceptions of the DMR service.
Nazar, H et al	British Medical Journal	Paper	2015	New transfer of care initiative of electronic referral from hospital to community pharmacy in England: a formative service evaluation.	Background information about the PharmOutcomes system. The effect of PharmOutcomes referrals on hospital readmission rates.
Wilcock, M et al	Pharmacoepidemiology and Drug Safety	Paper	2018	Growing the evidence base for transfer of care to community pharmacy?	Background information about the PharmOutcomes system. The effect of PharmOutcomes referrals on hospital readmission.
Ferguson, J et al	International Journal of Pharmacy Practice	Abstract	2016	Seamless transfer of medicines information from hospital to community: Implementation lessons from two case studies	Background information about the Refer-to-Pharmacy system. Hospital pharmacist perceptions of the implementation of Refer-to-Pharmacy.
Ferguson, J et al	BMC Health Services Research	Paper	2018	Refer-to-pharmacy: A qualitative study exploring the implementation of an electronic transfer of care initiative to	Background information about the Refer-to-Pharmacy system. Hospital pharmacy staff and community pharmacist perceptions of the Refer-to-Pharmacy system.

				improve medicines optimisation following hospital discharge	
Royal Pharmaceutical Society	Royal Pharmaceutica l Society	Report	2014	Hospital referral to community pharmacy: An innovators' toolkit to support the NHS in England	Description of exemplar examples of technology-supported transfer of care systems including PharmOutcomes, Refer-to-Pharmacy and Help for Harry.
Gray, Alistair	Pharmacy	Paper	2015	Refer-To-Pharmacy: Pharmacy for the Next Generation Now! A Short Communication for Pharmacy	A brief description of how Refer-to-Pharmacy works in practice.
Gray, Alistair	British Journal of Hospital Medicine	Paper	2015	Electronic referrals from hospital bedsides to community pharmacies	Background information about the need for transfer of care systems and an explanation of how Refer-to-Pharmacy presents a solution.
Hodson, K et al	FIP Congress	Abstract	2018	A four-year evaluation of the discharge medicines review service provision across all Wales	A description of DMR provision between 2014 and 2018

Table S2: Brief summary of literature gathered from the targeted literature search

Author	Publisher	Literature Type	Year	Title	Summary
The Eastern Academic Health Science Network	YouTube	Video	2018	PharmOutcomes	A demonstration of how to perform PharmOutcomes referrals from hospital to community.
Gray, A	YouTube	Video	2017	Refer-to-Pharmacy hospital pharmacy training film February 2017	An extensive demonstration of Refer-to-Pharmacy.
Gray, A	YouTube	Video	2016	Refer-to-Pharmacy hospital demo featuring the new Hospital Admission Notification message	A demonstration of the hospital admission notification system for Refer-to-Pharmacy.
Gray, A	Refer-to-Pharmacy	Webpage	2018	Refer-to-Pharmacy	A website dedicated to Refer-to-Pharmacy news and progress. Includes information on the background of Refer-to-Pharmacy and its philosophy.

Hodson, K et al	Community Pharmacy Wales	Report	2014	Evaluation of the Discharge Medicines Review service	A large report containing the service evaluation for the DMR service. It includes extensive background for the DMR service and stakeholder perceptions.
NHS Wales Informatics Service	Community Pharmacy Wales	Report	2018	Choose Pharmacy user guide version 7.0	A description of the process for the DMR referral system.
Leeson, N	Pharmaceutical Journal	Article	2018	Post-discharge medicines scheme is underutilised, study suggests	An article describing the underutilisation of the DMR service.
Gray, A	YouTube	Video	2017	Refer-to-Pharmacy Hospital Admission notification AND new outcomes capture Jan 2017	A demonstration of hospital admission notifications and the data capture functionality of Refer-to-Pharmacy.
Gray, A	YouTube	Video	2017	Refer-to-Pharmacy Community Pharmacy training film February 2017	A demonstration of how Refer-to-Pharmacy referrals are processed in community pharmacies.
Roberts, A	YouTube	Video	2017	PharmOutcomes instructional video	A demonstration of how to complete a PharmOutcomes referral in community pharmacy.
Staffs & Stoke Pharmacies	YouTube	Video	2018	Pharmoutcomes introduction V2	A demonstration of how to perform PharmOutcomes referrals from hospital to community.
Pinnacle Health Partnership	PharmOutcomes	Webpage	2018	PharmOutcomes. Delivering evidence	A website dedicated to the PharmOutcomes portal. Information included about the history and provision of PharmOutcomes and user-support guides.
Pinnacle Media	PharmOutcomes	Webpage	2018	PharmOutcomes Media	Multiple videos demonstrating how to perform a referral through PharmOutcomes and how to action a referral in community pharmacy.

PSNC	PSNC	Webpage	2018	Medicines Use Review	Description of the MUR service including its use post-discharge
PSNC	PSNC	Webpage	2018	New Medicines Service	Description of the NMS service including its use post-discharge
